# Insights Into the Links Between Proteostasis and Aging From *C. elegans*

**DOI:** 10.3389/fragi.2022.854157

**Published:** 2022-03-18

**Authors:** William Hongyu Zhang, Seda Koyuncu, David Vilchez

**Affiliations:** ^1^ Cologne Excellence Cluster for Cellular Stress Responses in Aging-Associated Diseases (CECAD), University of Cologne, Cologne, Germany; ^2^ Center for Molecular Medicine Cologne (CMMC), University of Cologne, Cologne, Germany; ^3^ Faculty of Medicine, University Hospital Cologne, Cologne, Germany

**Keywords:** proteostasis, *C. elegans*, protein translation, chaperones, ubiquitin-proteasome system, autophagy, protein aggragation, stress responses

## Abstract

Protein homeostasis (proteostasis) is maintained by a tightly regulated and interconnected network of biological pathways, preventing the accumulation and aggregation of damaged or misfolded proteins. Thus, the proteostasis network is essential to ensure organism longevity and health, while proteostasis failure contributes to the development of aging and age-related diseases that involve protein aggregation. The model organism *Caenorhabditis elegans* has proved invaluable for the study of proteostasis in the context of aging, longevity and disease, with a number of pivotal discoveries attributable to the use of this organism. In this review, we discuss prominent findings from *C. elegans* across the many key aspects of the proteostasis network, within the context of aging and disease. These studies collectively highlight numerous promising therapeutic targets, which may 1 day facilitate the development of interventions to delay aging and prevent age-associated diseases.

## 1 Introduction

The use of short-lived, rapidly reproducing, and easily modified model organisms has allowed us to undertake cause and effect studies for cellular and organismal aging on a massive scale. The nematode *Caenorhabditis elegans* in particular provides exceptional utility, as it possesses high genetic homology with humans (>70%), and conserved biological signaling pathways (S. Zhang et al., 2020). With the aid of *C. elegans* and other model organisms, we now know that many age-associated phenotypes do not depend on the chronological age of an organism, but instead depend on the accumulation of damage to the genome or proteome, and are defined by key signaling cascades such as the insulin/insulin-like growth factor (IGF-1) signalling (IIS) pathway (López-Otín et al., 2013). Identifying and exploiting biological networks or molecular targets that control organism aging and longevity has thus become the focus of research, with the long-term goal of translating these findings into therapeutic strategies. This is similarly true for the study of diseases arising from protein dysregulation, where *C. elegans* is an invaluable model towards the study of neurodegenerative proteinopathies, and often within the context of aging (Saez and Vilchez, 2014; Koyuncu et al., 2015).

Protein homeostasis (proteostasis) is crucial for organism longevity and health, and impairment to the proteostasis network is a hallmark of aging (López-Otín et al., 2013). This network principally functions to maintain proteome integrity, and is inclusive of the processes encompassing the translation and post-translational processing of newly-synthesized proteins, as well as those that control protein localization and degradation (Hipp et al., 2019). However, during aging this network becomes progressively impaired, and this drives the accumulation of misfolded, dysfunctional and aggregated proteins (Saez and Vilchez, 2014).

**FIGURE 1:**
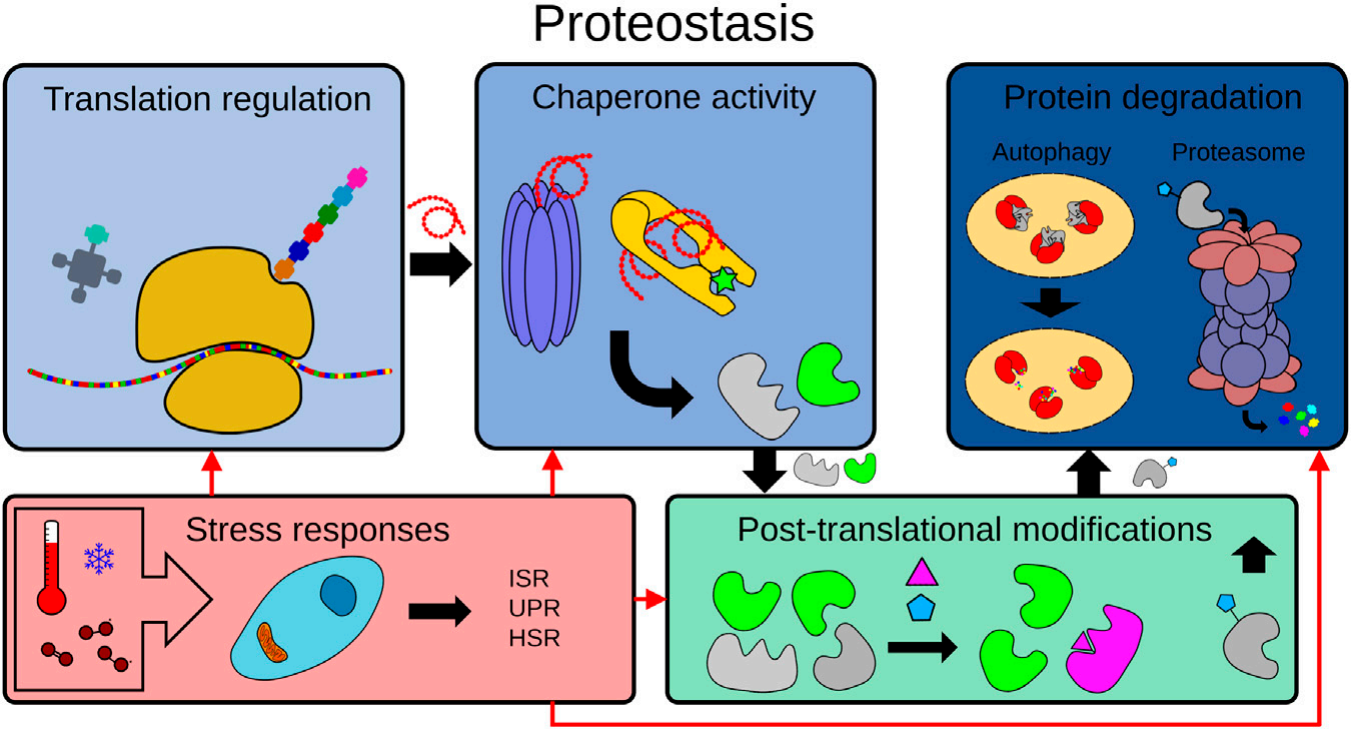
The proteostasis network. To maintain proteome integrity, the proteostasis network is tightly regulated in healthy cells from translation to degradation. The proteostasis network includes protein translation, protein folding by chaperones, post-translational modifications (PTMs), protein degradation mechanisms (e.g. the ubiquitin-proteasome system (UPS), the autophagy-lysosome pathway) and the modulation of adaptive stress responses by physiological or environmental signals (e.g. ISR: integrated stress response, UPR: unfolded protein response, HSR: heat shock response.

Here, we discuss how *C. elegans* has been used to understand and exploit the underlying mechanisms behind proteostasis in determining organismal longevity and aging. This will encompass discussing protein translation, folding and maintenance by chaperones, post-translational modifications, and the two main proteolytic mechanisms: the ubiquitin-proteasome (UPS) and the autophagy-lysosome pathway (Figure 1).

## 2 Protein Synthesis

**FIGURE 2:**
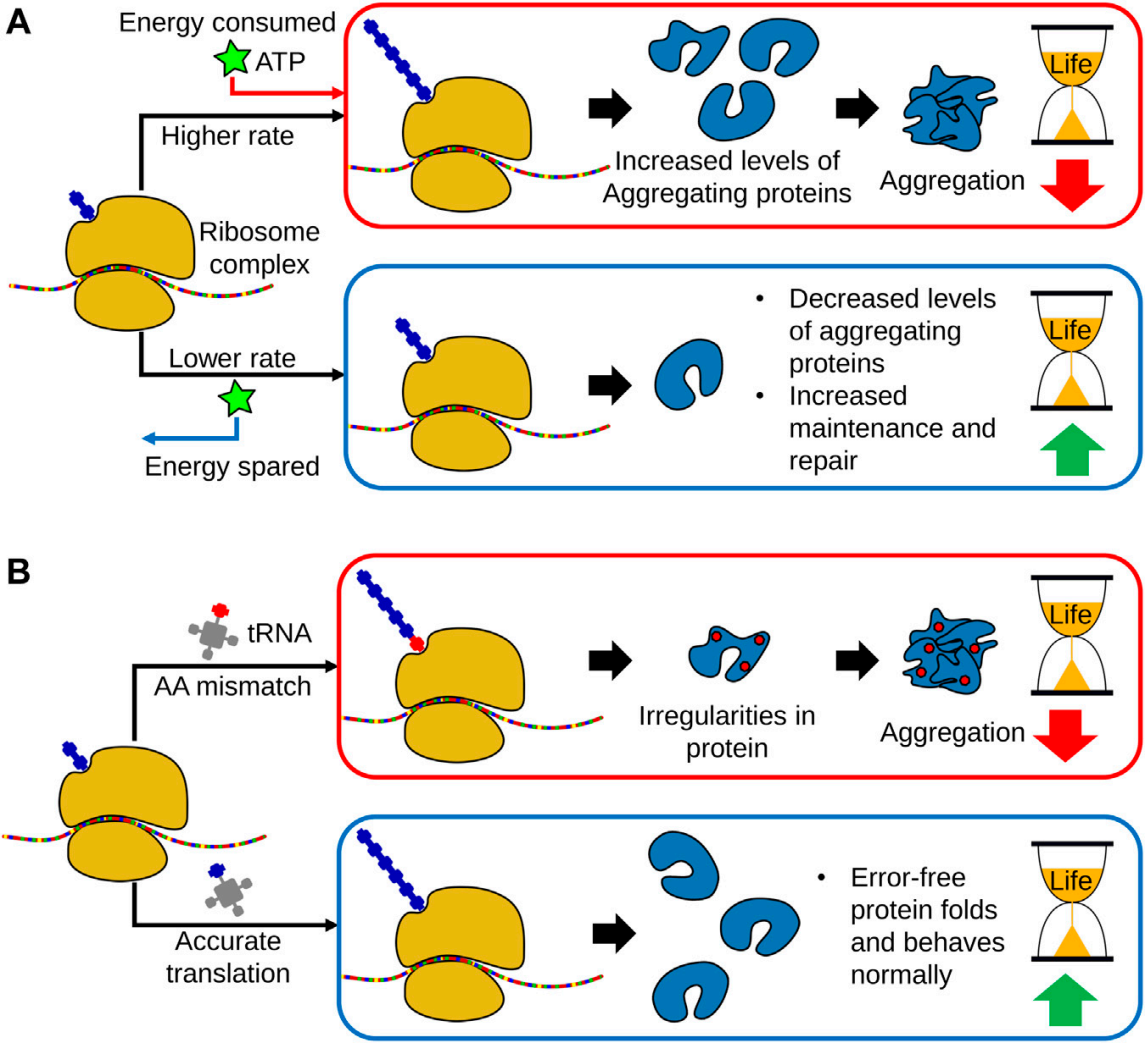
The links between translational regulation and aging. **(A)** With age, global translational rates decrease in a broad range of organisms, indicating a link between loss of protein synthesis capacity and aging. However, translation is a highly energy consuming process and growing evidence indicates that a decline in protein synthesis allows a greater allocation of metabolic energy towards cellular maintenance and repair. Subsequently, a decrease in protein translation promotes proteostasis and leads to lifespan extension. Moreover, lowering protein translation also decreases the production of aggregation prone-proteins. **(B)** In addition to global translational rates, translational fidelity may also decline with age, leading to increased amino acid misincorporation, as well as erroneous start and stop codon readthrough. This results in the production of dysfunctional proteins and peptides, thereby increasing the accumulation of protein aggregates. As such, accurate translation is necessary for organismal homeostasis and long lifespan.

Both the overall rate of protein synthesis and the fidelity of translation decreases with age. These two observations are distinct and reproducible across numerous studies and model organisms (Anisimova et al., 2018). However, genetic modulation to induce a decreased rate of protein synthesis has been shown to be generally lifespan-extending, while conversely, a decrease in translational accuracy is associated with aged and diseased phenotypes (Syntichaki et al., 2007b; Anisimova et al., 2018; Martinez-Miguel et al., 2021) (Figure 2). As such, these two parameters of protein synthesis are often studied independently from each other, and rely on different biological components and mechanisms for their observed effects on lifespan.

### 2.1 Translational Rates

Aging causes a functional decline in various components of the protein translation system, as well as age-related regulatory changes (Walther et al., 2015; Dhondt et al., 2017; Anisimova et al., 2018). The net effect is that the rate and the frequency of protein translation, and by extension protein synthesis, decreases with age, with many of these age-associated changes often having detrimental impacts on health and lifespan (Syntichaki et al., 2007b; Dhondt et al., 2017). This observed correlation has previously led to the speculation of a possible relationship between a decreased translation rate and the progression of age (Tavernarakis and Driscoll, 2002). However, contrary to this expectation, a number of studies have since shown that decreased protein synthesis increases lifespan in both normal aging and long-lived paradigms (Syntichaki et al., 2007b; Depuydt et al., 2016; Dhondt et al., 2017). Thus, studies regarding protein translational rates focus not only on the characterization and understanding of translation pathways and regulators in respect to health and lifespan, but also on how these pathways can be exploited for potential therapeutic benefit.

There are primarily two non-conflicting theories often put forward to rationalize the lifespan improvement observed with the inhibition of protein synthesis. The first is that a decrease in protein synthesis allows a greater allocation of metabolic energy towards cellular maintenance and repair. This is broadly in line with the principles of the disposable Soma theory of aging (Jin, 2010), which postulates that there is a fitness cost in the growth and development of an organism through the diversion of resources away from cellular maintenance (Wieser and Krumschnabel, 2001; Anisimova et al., 2018). The second theory is that decreased protein synthesis also broadly reduces the expression of aggregation-prone proteins, thereby partially alleviating the buildup of toxic aggregates and reducing the burden placed on aggregate-clearance pathways (Silva et al., 2011; Kim and Strange, 2013; Solis et al., 2018). From a molecular perspective, there are a wide array of causes behind this decrease in protein synthesis. This includes a broad decrease in transfer RNAs (tRNAs) availability, downregulation and functional impairment of ribosomes, as well as several hormonal and transcriptional changes (Gonskikh and Polacek, 2017).

#### 2.1.1 The Mechanistic Target of Rapamycin Pathway

One of the most crucial, and rate limiting, steps of translation is the initiation of translation by the mTOR pathway (Johnson et al., 2013). This pathway, which is itself regulated by the availability of metabolic energy and resources, controls various aspects of growth, development, metabolism and stress responses. As such, it possesses tight control over protein synthesis, controlling the initiation and inhibition of key genes. The two different mTOR complexes, mTORC1 and mTORC2, are the central regulatory components of this pathway, with each containing the mTOR kinase as the core functional unit (Johnson et al., 2013). Our understanding of this pathway and it is constituent components in the context of lifespan and aging, while far from complete, has been greatly bolstered by numerous studies utilizing *C. elegans*. We know that downregulation of the mTOR pathway leads to an extension of organismal lifespan, where, as discussed previously, this negative regulation leads to a reduction in protein translation. This has been demonstrated through RNA interference (RNAi) mediated knockdown of various *C. elegans* analogues of mTOR pathway components, such as the mTOR kinase (*let-363*), or the mTORC1 component raptor (*daf-15*), which was found to yield an increase in lifespan (Vellai et al., 2003; Jia et al., 2004). The pharmacological inhibition of mTOR activity by rapamycin similarly results in an extension of lifespan (Cornu et al., 2013). Caloric restriction also increases lifespan through the downregulation of mTOR activity. Likewise, reduced insulin signaling, achievable through calorie restriction, leads to the activation of glutamine synthase (GS), which then inhibits mTORC1 activity (van der Vos et al., 2012). Moreover, the insulin-regulated transcription factor DAF-16 (a FOXO analogue) negatively regulates transcription of *daf-15* and therefore the mTOR pathway (Jia et al., 2004). Interestingly, mTOR inhibition through caloric restriction also upregulates autophagy, another important component of the proteostasis network (Hansen et al., 2008; Tóth et al., 2008). Collectively, these studies in *C. elegans* demonstrate that the mTOR pathway is broadly essential for longevity regulation through cross-talk with other pathways, and also tightly regulates translation. However, they also highlight important caveats, where although inhibition of the mTORC1 complex can enhance lifespan, it is not without undesirable “side effects”. This includes delayed development, metabolic impairment, and decreased fertility (Schreiber et al., 2010; Zhang et al., 2019). Furthermore, some of these changes in metabolism and development may not be true “side effects”, but are rather a direct outcome of lifespan extension arising from the fitness cost in diverting energy and resources away from the growth and development of organism towards maintenance (Blagosklonny, 2013; Maklakov and Chapman, 2019). Notably, recent studies have shown that neuronal mTORC1 activation promotes lifespan extension without delayed development (Zhang et al., 2019; Smith et al., 2021) and another study found that reduced translation in neurons, hypodermis or germline through inhibition of mTOR pathways improved survival rate, suggesting that mTOR inhibition regulates lifespan in a tissue-dependent manner (Howard et al., 2021). These studies in *C. elegans* thus not only advance our understanding of the mTOR pathway in organism development and proteostasis maintenance, but also provide important insights into the utility of the mTOR pathway as a potential therapeutic target in humans for combatting aging.

#### 2.1.2 Translation Factors: Ribosomes, Elongation Factors, and Initiation Factors

Outside of the mTOR pathway, translation rate is determined by the combined interactions between the different components of the translational machinery. As such many studies have focused on individual components to assess their role and importance to translation in the context of aging. RNAi-mediated knockdown of components of either the small or large ribosomal subunits prolongs lifespan in *C. elegans* (Hansen et al., 2007). Moreover, depletion of the translational regulator S6K similarly extends lifespan while also decreasing age-associated protein aggregation (Hansen et al., 2007; Yee et al., 2021). Likewise, inhibition of distinct translation initiation factors also enhances *C. elegans* lifespan by up to 50% (Curran and Ruvkun, 2007). It has been shown that depletion of IFE-2, a worm orthologue of human eIF4E, increases lifespan through decreasing global protein synthesis (Syntichaki et al., 2007a). Interestingly, although IFE-2 availability has been known to decline with age, inhibition of this factor resulted in improvements to the lifespan and stress resistance of *C. elegans* (Hansen et al., 2007; Syntichaki et al., 2007a). Moreover, another study has shown that IFE-2 is highly sequestered in mRNA processing (P) bodies due to age and upon stress, and this sequestration decreases translation in somatic tissues (Rieckher et al., 2018). Depletion of another initiation factor, eIF4G*/ifg-1*, and deletion of two distinct subunits of eIF3 also increases lifespan through decreasing protein synthesis (Pan et al., 2007; Rogers et al., 2011; Cattie et al., 2016). Cumulatively, these studies support the principle that decreasing protein synthesis improves lifespan, furthermore they have identified key translation initiation factors that appear to be primary mediators of organismal longevity.

Indeed, translation initiation factors are central components of survival and stress responses. One such example is the integrated stress response (ISR), an important signaling pathway for the regulation of protein translation that relies on the phosphorylation of the translation initiation factor eIF2 (Derisbourg et al., 2021a). Interestingly, inhibition of the ISR through mutations to the eIF2-activating protein complex eIF2B promotes proteostasis and enhances lifespan (Derisbourg et al., 2021b). Similarly, preventing the phosphorylation of the eIF2α subunit of the eIF2 complex by either mutations or pharmacological inhibition increases lifespan. However, this extension was found to not be due to a decline in overall protein synthesis, but instead arises from variations in the translational efficiency of a subset of mRNAs (Derisbourg et al., 2021b). The downstream effects of the ISR have a crucial role in the proteostasis network for organism survival, and is discussed in-depth further in this review. Nonetheless, these studies indicate that decreasing protein synthesis for a subset of genes, rather than a global decrease to protein synthesis, may be sufficient to achieve an improvement to lifespan. Furthermore, such studies indicate that because these translational elements are involved in different stress responses, they can also influence organismal lifespan by mechanisms besides resource preservation arising from global protein synthesis inhibition.

Collectively, growing evidence supports that decreasing the rate of protein synthesis can have pro-longevity effects, with the majority of proposed mechanisms relying on the reduction of cellular burden, either metabolically or proteopathically. These insights have been made possible through the use of *C. elegans* as a model organism, and although comparatively slower, these key findings are starting to be reproduced in mammalian models (Essers et al., 2016; Thompson et al., 2016; Swovick et al., 2021).

### 2.2 Translation Fidelity

Although there is ongoing debate as to how significantly translation fidelity changes with age, cumulative evidence firmly indicates that the accurate synthesis of proteins defines organismal lifespan and is essential for organismal health (Tavernarakis and Driscoll, 2002; Anisimova et al., 2018; Ke et al., 2018; Francisco et al., 2020). Excluding genomic causes, these errors primarily arise due to inaccurate start and stop codon recognition by the ribosome, amino acid mismatch during the aminoacylation of tRNAs by aminoacyl-tRNA synthetases, and inaccurate aminoacyl-tRNA selection by the ribosomes. One study showed that the lifespan of *C. elegans* could be improved by increasing translational fidelity, through the use of pharmacological anti-aging treatments such as rapamycin, trametinib and torin 1 (Martinez-Miguel et al., 2021). This study further demonstrated that a fidelity-improving mutation to the ribosomal 40S subunit RPS23 could likewise improve lifespan primarily by decreasing erroneous stop-codon read-through, with this lifespan extension reproducible across multiple species, including *C. elegans*. Crucially, this mutation did not impact the rate of translation, allowing translational accuracy to be studied in a manner isolated from the effects on translation rate (Martinez-Miguel et al., 2021). Another study showed that knocking out *efk-1*, the *C. elegans*, orthologue of the elongation factor eEf2K, decreases translation fidelity and lifespan (Xie et al., 2019). Furthermore, depletion of multiple aminoacyl-tRNA synthetases (ARSs) including leucyl, arginyl, asparaginyl and methionyl ARSs similarly caused a decrease in lifespan through higher levels of amino acid misincorporation (Xie et al., 2019). It has also become increasingly apparent that errors in protein translation may arise from faulty RNA splicing, where it has been shown that RNA splicing fidelity decreases during aging, and that this decline is ameliorated by caloric restriction (Heintz et al., 2017). In addition, the exposure to reagents such as cadmium lead to disruption in RNA splicing and contribute to aging (Wu et al., 2019).

Other studies in *C. elegans* have found that inhibition of some ARS can instead have pro-longevity effects. In these lines, RNAi-mediated knockdown of the tyrosine ARS *yars-2* is necessary for the longevity phenotype of *daf-2* mutant worms, a genetic model of reduced IIS signaling (Son et al., 2017). Similarly, another study showed that the leucine ARS *lars-1* activates the mTOR pathway, which as discussed previously, negatively impacts lifespan (Nakamura et al., 2016). These effects on lifespan appear to be independent of changes to translational fidelity, and are a relatively recent discovery. As such, much of the regulatory roles played by ARSs and tRNAs remains unclear. Indeed, despite evidence that the misaminoacylation of tRNAs can lead to diseases that involve protein aggregation, misaminoacylation has recently been acknowledged to play an important functional role in various cellular processes and stress responses (Schimmel, 2018). Although studies investigating the role of misaminoacylation in aging and disease with *C. elegans* remains sparse, there are an increasing number of studies using *C. elegans* to study how ARSs and the loss of tRNA into small tRNA-derived fragments contributes to aging and disease, with detailed studies and extensive reviews into this subject available elsewhere (Kato et al., 2011; Shin et al., 2021; Zhou et al., 2021).

From studies in *C. elegans*, there is now clear evidence that translational fidelity is important for the preservation and maintenance of proteome integrity, and by extension both longevity and healthspan. Moreover, these studies identified new sources of translational error, and have begun to clarify previously unknown regulatory roles of the different components of the translational machinery. These new findings may have significant consequences for therapeutic development, and as such, there is ample room for further research.

### 2.3 Protein Folding

**FIGURE 3:**
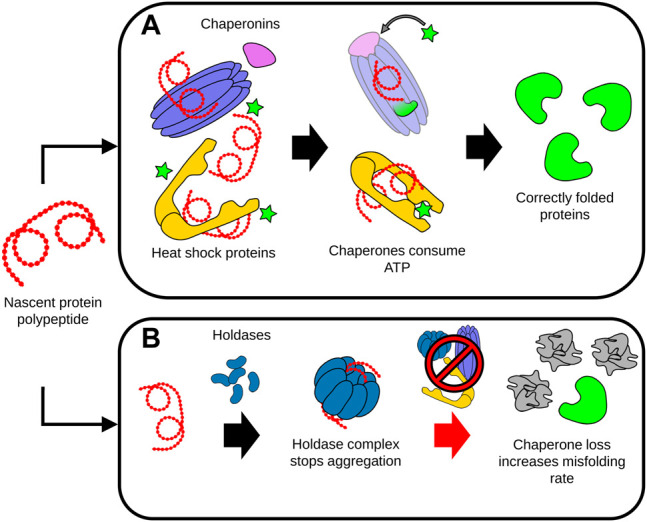
Dysregulation of protein folding during aging. **(A)** Chaperones assist the proper folding, refolding and disaggregation of proteins. ATP-dependent chaperones such as chaperonins and heat shock proteins interact with protein polypeptides to stabilize folding intermediates. The energy provided by ATP enables the conformational support and subsequent release of the folded protein by the chaperone complexes. **(B)** Unfolded proteins or polypeptides can be sequestered by holdases. Holdase proteins assemble into higher-order complexes, capable of isolating and preventing the aggregation of the disordered unfolded proteins and peptides. During aging, deficits in chaperone levels and activity significantly increases the rate of protein misfolding, accelerating the accumulation of damaged, misfolded and aggregated proteins This accumulation of misfolded proteins can in turn overwhelm the capacity of the remaining chaperones to maintain proteostasis, leading to cell malfunction and death.

The chaperome network is formed by chaperones and co-chaperones that have an integral role in enabling the assembly of proteins into a functional state (Brehme et al., 2014). As such, the chaperome network stabilizes folding intermediates of newly synthesized or unfolded proteins, and further prevents the denaturation or irreversible aggregation of many proteins (Heintz et al., 2017). Chaperones can be broadly classified as ATP dependent, or ATP independent (Mogk et al., 2018). ATP-dependent chaperones such as HSP70 are responsible for both protecting proteins against aggregation while also using the chemical energy provided by ATP to overcome thermodynamically unfavorable intermediates during folding. ATP-independent chaperones such as small heat shock chaperones, also called holdases, likewise bind to unfolded proteins to prevent their aggregation, but most are not thought to contribute to protein folding (Mogk et al., 2018). Due to their essential role, chaperones must be ever-present in cells for proteome maintenance and protein folding. However, with age, misfolded and aggregated proteins accumulate and exceed the stabilizing capacity of available chaperones, diverting many chaperones away from other crucial functions such as regulating the proper folding of nascent proteins (Labbadia and Morimoto, 2015) (Figure 3). In addition, there is evidence from studies in *C. elegans* showing that chaperone expression becomes increasingly impaired with age, further compounding the burden of insults to the proteome (Labbadia and Morimoto, 2015).

The overexpression of several chaperones, such as small heat shock proteins and members of Hsp70 family, decreases aggregate formation and extends lifespan in *C. elegans* (Yokoyama et al., 2002; Walker and Lithgow, 2003; Morley and Morimoto, 2004). Accordingly, the disruption of chaperone complexes can be deleterious to lifespan and health. Indeed, the TRiC/CCT complex, a chaperonin that promotes the folding of 10% of the proteome, is essential for regulation of longevity (Noormohammadi et al., 2016). Disruption in the assembly of TRiC/CCT complex causes cellular defects, while increasing TRiC/CCT assembly through the overexpression of the subunit CCT8 leads to an extended lifespan in *C. elegans* and reduces the neurotoxicity of aggregation-prone polyglutamine (polyQ) (Noormohammadi et al., 2016). Accordingly, one paradigm is that protein aggregates are invariably deleterious to organism health and lifespan. However, this has been challenged by the proteomic analysis of long lived *daf-2* mutant worms, which found that long-lived adult *daf-2* worms possess a higher chaperone-associated aggregate load compared to wild-type worms (Walther et al., 2015). These aggregates were comparatively chaperone-rich, and were proposed to be a feature of a “protective aggregation response” that sequestered surplus and dysfunctional proteins in order to alleviate the burden to the proteostasis system. Interestingly, while there were no changes in Hsp70 and Hsp90 expression, small heat shock protein levels increased significantly, meaning this response prioritized sequestration and isolation of unfolded proteins, rather than repair and folding (Walther et al., 2015). Although there are several studies in other organisms that investigate the protective potential of aggregation (Saad et al., 2017), further work is required to fully characterize this potential stress response and how it impacts aging.

It has also become increasingly apparent that individual chaperones possess important regulatory functions outside protein stabilization and folding, and that these unique functions are not compensated for by other types of chaperones. For instance, RNAi-mediated knockdown of *daf-21*/Hsp90 chaperone in non-neuronal tissues decreases lifespan in both wild-type and long-lived *daf-2* worms. Notably, this lifespan attenuation revealed that Hsp90 ensures DAF-16 isoform A nuclear translocation and function, but this process does not rely on any of the protein stabilizing properties of Hsp90 (Somogyvári et al., 2018). Although there is much more to uncover in regards to other functions of individual chaperones, it is clear that chaperones are primary mediators of proteostasis and therefore lifespan. Studies in *C. elegans*, have begun to uncover, but also explore their role as the primary effector of numerous stress responses.

## 3 Post-Translational Modifications

**FIGURE 4:**
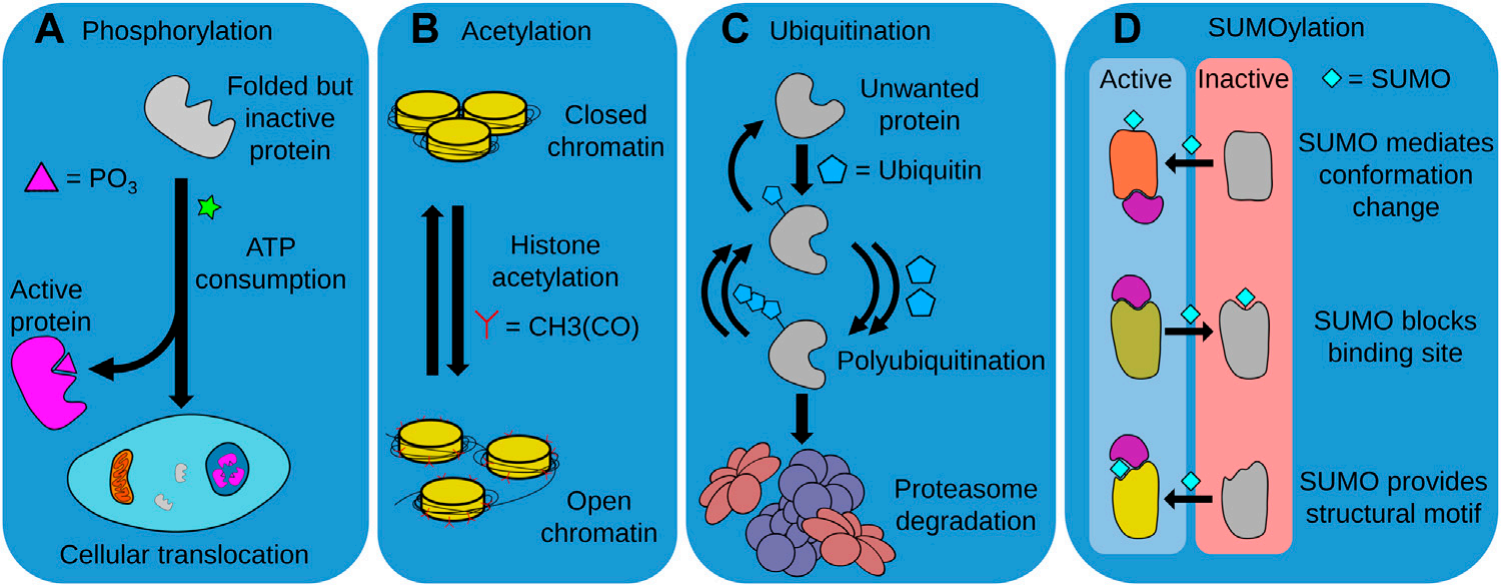
Post-translational modifications (PTMs). Distinct PTMs such as phosphorylation, acetylation, ubiquitination, and SUMOylation modulate the activity, intracellular localization and degradation of numerous proteins, determining cellular function and organismal longevity. **(A)** The ATP-consuming process of phosphorylation is required for the activity of many proteins, either through providing a functional chemical moiety, or by allowing the protein to translocate to the required cellular compartment. **(B)** Acetylation is also required for the function of various proteins, and is particularly important for correct chromatin function. In this instance, histone acetylation is required for chromatin opening and access to DNA by the cell. **(C)** The ubiquitination of unwanted proteins marks them for recognition and degradation by the UPS. This often requires repeated units of ubiquitin to be successively added to a growing polyubiquitin chain. The balance between ubiquitination and deubiquitination can thus control and regulate the composition of the proteome. **(D)** SUMOylation can both activate or deactivate modified proteins. SUMOylation can trigger conformational changes that allow proteins to interact with their biological substrates, block binding sites to prevent substrate interaction, or act as a component of a structural motif to enable recognition of the modified protein.

The regulation of protein fate through post-translational modifications (PTMs) is a crucial mechanism by which proteostasis is maintained. PTMs achieve this through several mechanisms, including modulation of protein stability, activity, and degradation (Ito et al., 2021). Although there are numerous PTMs, in this review, we focus on the four most common PTMs thought to be involved with aging and longevity, i.e. phosphorylation, SUMOylation, acetylation, and ubiquitination (Walsh et al., 2005) (Figure 4).

### 3.1 Phosphorylation

The addition of a phosphoryl group from adenosine triphosphate (ATP) to serine, threonine, or tyrosine residues serves as the most common PTM for proteins (Santos and Lindner, 2017). Phosphorylation is often necessary to facilitate functional conformational changes or provide a chemical moiety required for catalytic activity and protein-protein interactions (Yaffe, 2002). Numerous studies have demonstrated that protein phosphorylation regulates longevity in *C. elegans* (Derisbourg et al., 2021b; Huang et al., 2018; Lin et al., 2001; Li et al., 2021). One well studied mechanism of longevity regulation is in how phosphorylation controls various aspects of the IIS pathway (Kenyon et al., 1993; Lin et al., 2001). The foremost example arises when insulin-like ligands bind to the insulin/IGF-1 receptor DAF-2, which initiates a phosphorylation cascade culminating in the phosphorylation of the transcription factor DAF-16. Like its analogue FOXO in humans, the phosphorylation of DAF-16 results in its inactivation and retention in the cytoplasm, negatively regulating longevity by preventing the transcription of lifespan-extending effectors (Tatar et al., 2003). Targeted RNAi screens for different serine/threonine protein phosphatases to further study the role of the kinases in the IIS pathway have subsequently identified other novel longevity regulators, such as *pptr-1* (Padmanabhan et al., 2009). PPTR-1 negatively regulates the phosphorylation of AKT-1, preventing AKT-1 from inhibiting DAF-16. The modulation of *pptr-1* regulates a wide range of reduced IIS-related phenotypes including longevity, the activation of stress responses, and entry into the dauer state (Padmanabhan et al., 2009). A more recent study, similarly focused on IIS-dependent phosphorylation, demonstrated that there are 476 differentially regulated phosphosites in *daf-2* mutant worms (W.-J. Li et al., 2021). Their analysis also indicated that casein kinase 2 (CK2) negatively modulates longevity (Li et al., 2021).

These numerous studies thus cement phosphorylation as a fundamental component of the IIS pathway, however phosphorylation has also been found to play a crucial role beyond IIS. Phosphoproteomics analysis of *C. elegans* at two different temperatures (20 and 25°C) has revealed that phosphoprotein GTBP-1 modulates longevity at both temperatures, and promotes resistance to heat and oxidative stresses (Huang et al., 2018). This study also indicated that the kinases CK2, MAPK, and CAMK2 may similarly modulate aging through their kinase activity (Huang et al., 2018). Other studies have found that the phosphorylation of actin binding protein drebrin through the kinase ataxia-telangiectasia mutated (ATM) regulates lifespan and stress tolerance by improving the stability of drebrin and dynamics of actin remodeling (Kreis et al., 2019). Furthermore, phosphorylation of AMP-activated protein kinases has been found to be crucial for the regulation of cellular energy metabolism and cellular homeostasis, and by extension lifespan, across numerous studies (Hwang et al., 2014; Chang et al., 2017; Park et al., 2020). For example, one recent study demonstrates that the nuclear protein kinase vaccinia-related kinase (VRK-1) promotes lifespan extension through phosphorylation and activation of AMP-kinases (Park et al., 2020).

Thus, both preventing phosphorylation, as with DAF-16, or promoting phosphorylation, through phosphoproteins such as GTBP-1 or the phosphorylation of various AMP-kinases, can drastically increase lifespan, and firmly demonstrates that PTMs are key determinants of longevity.

### 3.2 Acetylation

Acetylation is the addition of an acetyl group to a nitrogen molecule of a target protein (Santos and Lindner, 2017). Numerous studies into the impact of protein acetylation on aging focus on age-associated changes in histone acetylation. During aging, histone acetylation is modulated, and is coupled to changes in metabolic activity and gene expression (Peleg et al., 2016). Sirtuin proteins are histone deacetylases, and act as important regulators of histone acetylation (Grabowska et al., 2017). Increased histone deacetylase activity of silent information regulator 2 (*Sir2*) extends lifespan (Tissenbaum and Guarente, 2001). Moreover, DAF-16 nuclear localization, which is essential for longevity and stress signal regulation, can be modulated by acetylation through the sirtuin SIR 2.4 protein (Chiang et al., 2012). Sirtuin activators such as oligonol also prolong lifespan in *C. elegans* infected with lethal *Vibrio* cholera (Park et al., 2016). Another study has indicated that early stage exposure to heat stress results in increased histone acetylation and helps the establishment of epigenetic “memory”, leading to an extended stress response and longevity in *C. elegans* (Zhou et al., 2019). Crucially, proteomics analysis comparing young worms to aged worms revealed the accumulation of acylated proteins, particularly in mitochondria (Hong et al., 2016). The accumulation of acylated proteins in mitochondria has been further shown to cause mitochondrial dysfunction and contribute to aging (Hong et al., 2016).

Although the detrimental effects of acetylated protein accumulation remains poorly understood, these studies nonetheless show that epigenetic and metabolic regulation by acetylation modifications have an important role in longevity.

### 3.3 SUMOylation

Protein SUMOylation is the covalent attachment of a small ubiquitin like modifier (SUMO) to lysine residues of target proteins (K. A. Wilkinson and Henley, 2010). This SUMOylation then facilitates, or prevents, the interaction of the modified protein with its interaction partner. SUMOylation is involved in several biological processes including development, DNA damage stress-responses, and mitochondrial dynamics (Flotho and Melchior, 2013). These diverse roles of SUMOylation are well studied, in particular, its role in development (Broday et al., 2004; Zhang et al., 2004; Kaminsky et al., 2009; Pelisch et al., 2017). However, many recent studies also highlight the role of SUMOylation in the regulation of longevity (Moll et al., 2018; Princz et al., 2020). Of note is how reduced IIS can modulate protein SUMOylation, where IIS driven SUMOylation of the germline RNA binding protein CAR-1 was found to shorten lifespan in *C. elegans*. Conversely, the expression of a mutant CAR-1, one which cannot be SUMOylated, promotes enhanced proteostasis and lifespan extension (Moll et al., 2018). The role of SUMOylation in IIS was further expanded in a recent study showing that SUMOylation of DAF-16 regulates mitophagy and mitochondrial dynamics, affecting lifespan (Princz et al., 2020). Furthermore, the same study showed a tissue-specific dependency on SUMOylation, where the RNAi-mediated knockdown of the small ubiquitin-like modifier gene, *smo-1*, shortened lifespan, while the overexpression of *smo-1*, specifically in intestine tissue, was sufficient to extend lifespan (Princz et al., 2020). Moreover, SUMOylation also regulates essential biological processes for healthspan and lifespan (Lim et al., 2014; Baytek et al., 2021). SUMOylation modulates the UPRER in *C. elegans* by regulating calreticulin gene expression in an XBP-1-dependent manner (Lim et al., 2014). SUMOylation also regulates chromatin dynamics by regulating protein activity of the chromodomain factor MRG-1 (Baytek et al., 2021). SUMOylation targets have previously been identified through gene ontology analysis, with most of these SUMOlyated proteins playing a role in metabolism (Drabikowski et al., 2018), and indeed evidence has been found that SUMOylation plays a role under calorie restricted conditions, by modulating the NHR-49 transcription factor (Drabikowski, 2020). These studies demonstrate that SUMOylation plays a decisive role in the proteostasis network for determining aging and longevity in *C. elegans*, in particular through modulation nodes of the IIS. However, as the majority of SUMOylation targets remain poorly characterized or understood, further research is necessary before the true impact of SUMOylation can be appreciated.

### 3.4 Ubiquitination

Ubiquitination is the covalent conjugation of ubiquitin, a small highly-conserved protein, to a lysine residue or N-terminal methionine of a target protein (Yau and Rape, 2016). Ubiquitination is a multistep reaction reliant on the coordination of E1, E2, and E3 enzymes (Hochstrasser, 2006). The process begins with ubiquitin-activating enzyme (E1), which activates ubiquitin through an ATP-dependent mechanism. The activated ubiquitin then transfers to the ubiquitin-conjugating enzyme (E2). Finally, a specific ubiquitin-protein ligase (E3) mediates the attachment of ubiquitin from the E2 enzyme to the target protein (Hochstrasser, 1996). Conversely, deubiquitinating enzymes (DUBs) can remove ubiquitin molecules and thereby unmark proteins (K. D. Wilkinson, 2000).

Ubiquitination can determine the fate of a given protein in several ways; it can mark it for degradation by the proteasome or autophagy pathways, regulate their activity, modulate protein-protein interactions and intracellular localization (Hershko and Ciechanover, 1998). Ubiquitination thus has a central regulatory role across a wide range of biological process, including signal transduction, transcriptional regulation, the DNA damage response, and the immune response (Hershko and Ciechanover, 1998). Furthermore, ubiquitination also regulates various stress responses and can also influence protein aggregation (Brehme et al., 2014) (Vilchez et al., 2014; Koyuncu et al., 2018). There is ample evidence that ubiquitination plays a crucial role in aging and longevity (Li et al., 2007; Powers et al., 2009; Kevei and Hoppe, 2014; Tawo et al., 2017; Koyuncu et al., 2021), however the underlying mechanisms are only now becoming clearer. The ubiquitination of a target protein is primarily achieved by the activity of E3 ubiquitin ligases, and can be reversed with deubiquitinase enzymes (Hochstrasser, 2006). We now understand that several of these E3 ligases and deubiquitinases modulate longevity (W. Li et al., 2007; Mehta et al., 2009). Several E3 ligases have been reported as regulators of the IIS pathway. For instance, the E3 ubiquitin ligase CHIP regulates the levels of DAF-2 through its ubiquitination and degradation, which modulates longevity (Tawo et al., 2017). Downregulation of RLE-1 E3 ligases results in less polyubiquitination of DAF-16, and increased DAF-16 transcriptional activation which results in extended lifespan (W. Li et al., 2007). The WWP-1 E3 ligases are required for the regulation of lifespan under caloric restricted conditions (Carrano et al., 2009). The lifespan regulation by WWP-1 depends on E3 Ubiquitin ligase activity and also interactions with the E2 ubiquitin conjugating enzyme UBC-18 (Carrano et al., 2009). Moreover, it has been shown the interactions of ubiquitin-selective chaperone CDC-48 and ATX-3 deubiquitinase modulate longevity through IIS (Kuhlbrodt et al., 2011). A decrease in both *cdc-48.1* and *atx-3* enhances their substrate stability and longevity by up to 50% (Kuhlbrodt et al., 2011). Recently, we have explored the role of ubiquitination in maintaining proteostasis and regulating longevity by analyzing system-wide ubiquitination changes that occur during aging (Koyuncu et al., 2021). Our findings revealed a global loss of ubiquitination during aging, which is ameliorated by longevity pathways, such as caloric restriction and reduced IIS (Koyuncu et al., 2021). This remodeling of ubiquitination patterns throughout the proteome is a result of elevated deubiquitinase activity. Remarkably, dysregulation of ubiquitination leads to the selective accumulation of various proteasome targets such as the intermediate filament, IFB-2 and EPS-8, a modulator of RAC signaling (Di Fiore and Scita, 2002; Geisler et al., 2019; Koyuncu et al., 2021). Accumulation of IFB-2 leads to loss of intestinal integrity while increased EPS-8 hyperactivates RAC signaling in muscle and neurons causing changes in actin cytoskeleton and hyperactivation of protein kinase JNK. Therefore, the dysregulation in the ubiquitination of structural and regulatory proteins across tissues contributes to aging features and regulate longevity (Koyuncu et al., 2021).

Ubiquitination thus has a much more significant role in aging than previously thought, and further research may allow us to therapeutically exploit parts of the ubiquitination network for anti-aging purposes.

## 5 Protein Degradation Systems

### 5.1 The Ubiquitin-Proteasome System

The UPS is the main system for selective degradation of proteins, determining the half-life of multiple regulatory proteins and controlling the clearance of damaged and unnecessary proteins (Pickart, 2001). Repeated addition of ubiquitin creates a polyubiquitin chain, marking the target protein for recognition and processing by the proteolytic machinery of the UPS, the 26S proteasome (Glickman and Ciechanover, 2002). A Lys48-linked polyubiquitin chain is the primary signal for recognition and degradation by the 26S proteasome. The 26S proteasome itself is composed of a 20S core catalytic particle and 19S regulatory particles (Saez and Vilchez, 2014).

The UPS declines after development in *C. elegans*, as observed by *in vivo* imaging strategy following the levels of chimeric green fluorescent protein fused to a non-cleavable ubiquitin moiety (Segref et al., 2011). Studies into the relationship between proteasome activity and longevity have shown that elevated UPS activity, mediated by elevated assembly or activity through induction of proteasome subunits, leads to an extension in lifespan (Vilchez et al., 2012; Chondrogianni et al., 2015). Reverting this age-related decrease in proteasome activity through the overexpression of 19S proteasome subunit *rpn-6.1* increased survival rate and heat stress resistance (Vilchez et al., 2012). Moreover, long-lived *glp-1* mutants, which lack a germline, have enhanced proteasome activity upon DAF-16 activation (Vilchez et al., 2012). Another study has shown that overexpression of the *psb-5* catalytic subunit of the 20S proteasome results in an extension of lifespan and resistance to oxidative stress in a DAF-16 dependent manner (Chondrogianni et al., 2015).

Similarly, epidermal growth factor (EGF) signaling has been reported to positively modulate proteasome activity by upregulating the expression of genes involved in the UPS such as Skp1-like protein SKR-5 (Liu et al., 2011). EGF signaling also regulates longevity by upregulating UPS activity, where animals lacking SKR-5 also have shorter lifespans (Liu et al., 2011). Other recent studies also support the role of proteasome activity on the regulation of lifespan, where defects in the import of mitochondrial proteins results in proteasome activation and lifespan extension (Sladowska et al., 2021).

These studies into the impact of proteasomal regulation on longevity highlights the importance of the UPS on aging and longevity. Thus, the proteasome can be an important target to find novel interventions to promote healthy aging.

### 5.2 Autophagy-Lysosome Pathway

Autophagy is an evolutionary conserved degradation pathway, in which cellular components including defective organelles and protein aggregates are sequestered in double-membrane vesicles and delivered to the lysosome for degradation (He and Klionsky, 2009). More than 30 proteins (encoded by *ATG* genes) are recruited at different steps of the autophagy process (Klionsky et al., 2016). There are three well-characterized types of autophagy: macroautophagy, microautophagy and chaperone-mediated autophagy (CMA). In macroautophagy, cytoplasmic components are first encapsulated into a double membrane-bound vesicle, called an autophagosome. This autophagosome then fuses with the lysosome to form an autolysosome, in which the sequestered cytoplasmic cargo is degraded with hydrolases, glycosidases, nucleotidases, lipases and proteases (Klionsky et al., 2016). In microautophagy, cytosolic cargo is delivered directly to the lysosome for degradation. In CMA, chaperones such as HSP70 promote the lysosomal degradation of targeted proteins (Glick et al., 2010).

Like the UPS, there is now substantial evidence that the autophagy-lysosome pathway is linked to the regulation of aging and age-related diseases. Dysfunctional autophagy during aging has been observed across a diverse range of species, including *C. elegans* (Aman et al., 2021). Genetic manipulation experiments of selective and non-selective autophagy pathway components have demonstrated an important role of autophagy in lifespan and healthspan (Tóth et al., 2008; Hansen et al., 2018; Kumsta et al., 2019). Either the knockdown or inactivating mutations of several autophagy components such as *bec-1* (orthologue of the mammalian APG6/VPS30/beclin1), *lgg-1, Igg-3, unc-51,* or *atg-7* leads to an accelerated aging phenotype and shortened lifespan (Tóth et al., 2008; Hansen et al., 2018). Moreover, several studies indicate that the upregulation of autophagy is mechanistically crucial for the extension of lifespan by different pro-longevity pathways (Meléndez et al., 2003; Hansen et al., 2008; Alberti et al., 2010; Gelino and Hansen, 2012). For instance, the RNAi-mediated downregulation of bec-1 suppresses the extension of lifespan in *daf-2* mutants (Meléndez et al., 2003). In addition, the knockdown of autophagy-regulating transcriptional factors such as *hlh-30* and *daf-16* shortens the lifespan of both wild type and *daf-2* mutant worms (Lin et al., 2018). It has been shown that worms exposed to dietary restriction have increased levels of the autophagy marker LGG-1 (the orthologue of ATG8) in their hypodermis, and require functional autophagy promoting genes for longevity (Mörck and Pilon, 2006; Hansen et al., 2008; Tóth et al., 2008).

Overexpression of the key regulator of autophagy *sqst-1/p62* induces autophagy in distinct tissues of *C. elegans*, leading to an extension of lifespan, and an overall improvement in organismal fitness (Kumsta et al., 2019). Overexpression of autophagy regulators such as AMPK enhances autophagy activity and extends lifespan (Hansen et al., 2018). Additionally, the induction of autophagy with different pharmacological agents such as spermidine, resveratrol, and metformin prolongs lifespan in *C. elegans* (Mariño et al., 2011; Gillespie et al., 2019). Pharmacological inhibition of XPO-1 similarly leads to increased autophagy and lifespan by nuclear enrichment of *HLH-30* in *C. elegans* (Silvestrini et al., 2018).

These studies consistently demonstrate that maintaining and upregulating autophagy can be beneficial for lifespan. Mechanistically, this longevity effects may arise from the persistent clearance of misfolded and aggregated proteins.

## 6 Adaptive Stress Response Mechanisms

Alterations to the activity of various organelles and biological pathways in response to stress has been extensively studied (Schulz et al., 2007; Dilberger et al., 2019; Taylor and Hetz, 2020; Derisbourg et al., 2021b). However, these stress responses are now known to change with age, and also contribute to organismal longevity. Given the stabilizing effects of chaperones, they are a key component of many adaptive responses to environmental stressors that challenge cellular integrity (Hipp et al., 2019). However, these stress responses also include wide regulatory changes, including variations in gene expression, activation of proteolytic pathways, and more. The most common stress responses in the cell include the heat shock response (HSR), the ISR, the unfolded protein response of the endoplasmic reticulum (UPR^ER^) and the UPR of the mitochondria (UPR^mt^). Here we focus on recent studies examining the link between the different stress responses and longevity in *C. elegans*.

### 6.1 Heat Shock Response

Proteotoxic stress, such as heat stress, can upregulate the expression of many specialist chaperones as a part of the HSR. Moreover, binding of damaged and misfolded proteins to chaperones leads to the liberation of heat shock transcriptional factors (HSFs) from chaperone complexes, which further upregulates the transcription of additional chaperones. The chaperones upregulated by the HSR principally act to stabilize and refold thermally denatured proteins, however the HSR also regulates a broad range of genes involved in normal aging, including the small heat shock protein *sip-1*, and *cyp-35B1*, a member of cytochrome P450 family (Hsu et al., 2003).

The HSR is mainly regulated by HSFs, specifically HSF-1 in *C. elegans* (Akerfelt et al., 2010). In worms, the activation of HSR by HSF-1 is also controlled by thermosensory neurons that sense temperature changes, in addition to the basal levels of HSF-1 present in chaperone complexes (Prahlad et al., 2008). Activation of HSF-1 induces the transcription of several chaperones, including HSP70, HSP90 family members, and small heat shock proteins (Hsu et al., 2003). There is substantial evidence that HSF-1 is also a regulator of aging, where HSR activation enhances longevity and stress tolerance by utilizing aspects of longevity-enhancing mechanisms similar to reduced IIS, caloric restriction and suppression of mTOR activity (Morley and Morimoto, 2004; Steinkraus et al., 2008; Seo et al., 2013; Kovács et al., 2019). Overexpression of *hsf-1* prolongs lifespan and decreases age-associated protein aggregation of disease-related proteins. Moreover, upregulation of HSF-1-target genes under unstressed conditions similarly extends longevity (Walker and Lithgow, 2003). Accordingly, RNAi-mediated knockdown of *hsf-1* shortens lifespan (Hsu et al., 2003; Morley and Morimoto, 2004). Interestingly, overexpression of a modified version of HSF-1, incapable of inducing the expression of HSPs, was found to also prolong lifespan (Baird et al., 2014). Thus HSF-1 can likely upregulate lifespan-extending genes outside what is considered the subset of HSR target genes. This is supported by other studies, where transcriptomics analysis revealed that several distinct longevity associated genes including *pha-4*, *lys-7* and *dod-3* are upregulated in HSF-1-dependent long-lived strains (Sural et al., 2019). Likewise, a recent study has also shown that mitochondrial stress can result in HSF-1 dephosphorylation, which induces the upregulation of lifespan extending holdases (Williams et al., 2020), further exemplifying the role of HSF-1 in mediating lifespan extending pathways. In addition, the upregulation of HSF-1 in neurons leads to the activation of DAF-16 in other tissues, making neuronal HSF-1 essential for longevity in a cell non-autonomous manner (Douglas et al., 2015).

Overall, studies in *C. elegans* have not only shown that the HSR is necessary for thermal protection of the proteome, but also for normal function under unstressed conditions. Moreover, HSF-1 is an essential modulator of aging and longevity through the activation of the HSR and other pathways.

### 6.2 Integrated Stress Response

The ISR is an important central stress response in eukaryotic cells, which is induced by a broad range of physiological and environmental changes (Pakos-Zebrucka et al., 2016). The ISR is primarily activated by the phosphorylation of a serine of eIF2*α*. This reaction is catalyzed by eIF2*α* kinases after stress stimuli such as viral infection, hypoxia and amino acid deprivation (Harding et al., 2003; Wek et al., 2006; García et al., 2007). The phosphorylation of eIF2*α* results in a broad decrease in protein translation, while increasing the translation of selected survival genes, such as activating transcription factor 4 (ATF4). If this adaptive response proves insufficient to counteract the stress, additional components of the ISR are activated to induce cell death, preventing potential cellular dysfunctions from impacting organismal health. After the stress stimulus disappears, or is mitigated, eIF2α is dephosphorylated, stopping the ISR and thereby allowing translation and other cellular process to return to normal levels (Novoa et al., 2003; Donnelly et al., 2013). In worms, there are two main eIF2α kinases; the general control nonderepressible 2 (GCN2) kinase, and the PKR-like endoplasmic reticulum kinase (PERK) (Derisbourg et al., 2021b). Previous studies have suggested that ISR is induced with age in different organisms (Derisbourg et al., 2021a), whereas enhanced ISR activation is already observed from early adulthood in *C. elegans* (Derisbourg et al., 2021b).

As discussed previously (section 2.1.2), although the decrease in protein synthesis induced by the ISR may be expected to be lifespan extending, the ISR is detrimental to lifespan in *C. elegans*. The cause behind this impairment to lifespan has only recently become to be understood. Through a large-scale mutagenesis screen, it was found that lifespan extending mutations to eIF2 inhibited the ISR, and these mutations relied on the putative kinase *kin-35*. Crucially, the lifespan extension mediated by kin-35 was found to be independent of any changes to protein synthesis (Derisbourg et al., 2021b). This finding indicates that the ISR may decrease lifespan due to the selective translation of key detrimental genes. Furthermore, contrary to previous studies that observed that knockouts of ISR kinases *gcn-2* and *pek-1* do not have impact on longevity, this study also showed that single inhibitory amino acid substitutions to GCN-2 and PEK-1 lead to lifespan extensions (Henis-Korenblit et al., 2010; Baker et al., 2012). There is still further investigation needed to fully understand the transcriptional changes caused by the ISR, and why this decreases lifespan in *C. elegans*.

The unfolded protein response of the endoplasmic reticulum (UPR^ER^).

The endoplasmic reticulum (ER) houses and regulates many of the chaperones that aid protein folding, as well as many enzymes that are responsible for the maintenance of proteostasis (Schönthal, 2012). The protein folding capacity of the ER is monitored by the unfolded protein response (UPR^ER^) signaling pathway. This pathway is conserved from yeasts to mammals, and is activated by the accumulation of unfolded and misfolded proteins in the ER lumen (Hipp et al., 2019). To maintain protein folding fidelity, the UPR^ER^ regulates mRNA translation to decrease the further accumulation of misfolded proteins, while also upregulating folding chaperones in the ER (Taylor and Hetz, 2020). In the metazoan ER, there are three identified activators for the different signaling pathway sub-branches of the UPR^ER^, i.e. IRE1 (inositol-requiring enzyme), PERK (protein kinase RNA-like endoplasmic reticulum kinase), and ATF6 (activating transcription factor 6).

Activation of the UPR^ER^ by external stress declines with age, and this decline is associated with several age-related diseases (Taylor and Dillin, 2011). By studying the metazoan UPR^ER^-component analogues in *C. elegans*, we have begun to understand what contributes to this decline in UPR^ER^ responsiveness, and how this impacts organismal aging. Genetic manipulation of the ER stress response has shown that the UPR^ER^ has an important role in the modulation of lifespan and healthspan (Klionsky et al., 2016). Further studies in *C. elegans*, demonstrated that overexpression of the stress-activated transcriptional factor XBP-1 in neurons prolongs lifespan in a cell non-autonomous manner (Taylor and Dillin, 2013). In addition to neuronal UPR^ER^ activation, intestinal UPR^ER^ activation enhanced longevity through an increase in lipophagy (Imanikia et al., 2019; Daniele et al., 2020). A recent study further demonstrated that constitutive activation of the UPR^ER^ with XBP-1 in astrocyte-like glia prolongs lifespan and stress resistance (Frakes et al., 2020). Moreover, a wide range of longevity paradigms such as reduced insulin signaling and caloric restriction require the UPR^ER^ (Chen et al., 2009; Henis-Korenblit et al., 2010; Matai et al., 2019). Thus, the consensus between these studies is that activation of the UPR improves lifespan, and is most likely mediated through the increased presence of chaperones preventing aggregate formation, as well as some contribution from upregulation of various metabolic processes such as lipophagy.

Interestingly, although the UPR^ER^ is not activated in the long-lived *daf-2* mutant worms, an enhancement to lifespan through reduced insulin signaling requires the presence of IRE1α/XBP1 (Henis-Korenblit et al., 2010). Conversely, caloric restriction of worms induces a higher basal level of UPR^ER^ activity, and the increased lifespan phenotype requires the ER stress branch IRE-1 (Matai et al., 2019). This provides clear evidence that the IIS and UPR^ER^ are in some way linked by a common regulatory element, and that the UPR^ER^ contributes to the lifespan enhancement observed under calorie restricted conditions. However, the precise mechanism behind how caloric restriction, or the IIS pathway, influence or utilize the UPR^ER^ remains unclear.

It has been also shown that the activation of the hexosamine pathway, which leads to enhanced UPR^ER^ activity, increases lifespan through improvement of ER-associated protein degradation (Denzel et al., 2014). Another study has also reported that enhancing lifespan with vitamin D treatments requires IRE1*α* and XBP1 (Mark et al., 2016). Additionally, treatment with activators of the UPR^ER^, such as tunicamycin, prolongs lifespan based on IRE1α branch of UPR^ER^ (Matai et al., 2019). Accordingly, mutations in IRE1α or XBP1 has been found to shorten lifespan (Taylor and Hetz, 2020). Collectively, these studies show that UPR^ER^ activation is necessary for both normal and enhanced organismal lifespan, that multiple regulatory and nutrient sensing pathways converge on the UPR^ER^, and also that various components of the UPR show promise as therapeutic targets for anti-aging outcomes.

The unfolded protein response of the mitochondria (UPR^mt^).

The mitochondria provides cellular energy and regulates a broad range of metabolic events (Dilberger et al., 2019). As a consequence of the process of oxidative phosphorylation (OXPHOS), mitochondria produce reactive oxygen species (ROS) (Sies and Cadenas, 1985). Elevated levels of ROS can lead to cellular damage, with such damage also called oxidative stress (Sies and Cadenas, 1985). Impairment of the mitochondria is associated with cellular dysfunction and is considered a hallmark of aging. Damage to mitochondrial integrity induces transcriptional responses, including the mitochondrial unfolded protein response (UPR^mt^) which is regulated by mitochondrial-to-nuclear communication. The UPR^mt^ induces the recovery of mitochondrial networks through mitochondrial biogenesis and metabolic adaptations, promoting cell survival under stress conditions (Münch and Harper, 2016; Shpilka and Haynes, 2018). Whereas an acute stress in the mitochondria can lead to cell dysfunction and death, a reduced amount of mitochondrial stress can be beneficial for organismal longevity in a process known as mitohormesis. Mitohormesis also encompasses the activation of the UPR^mt^.

In *C. elegans*, the UPR^mt^ is regulated by the transcription factor ATFS-1 and the co-factors DVE-1 and UBL-5, where ATFS-1 is activated by disruptions to mitochondrial proteostasis as well as ROS produced by OXPHOS. This activation leads to transcriptional regulation to regulate survival and mitochondrial stress through ATFS-1 (Shpilka and Haynes, 2018). It is important to note that although ATSF-1-regulated genes are upregulated in long-lived worms, chronic expression of ATFS-1 itself is not sufficient to extend lifespan (Soo et al., 2021). However, activation of the UPR^mt^ is known to positively regulate longevity in *C. elegans* (Dillin et al., 2002; Durieux et al., 2011; Ito et al., 2021). In these lines, RNAi-mediated knockdown of mitochondrial OXPHOS components such as complexes I, III and IV promotes longevity through activation of the UPR^mt^ (Feng et al., 2001; Dillin et al., 2002). Decreasing mitochondrial protein translation by knockdown of mitochondrial ribosomal protein S5 (*mrps-5*) likewise extends lifespan through enhanced UPR^mt^ (Houtkooper et al., 2013). Furthermore, changes in mitochondrial dynamics due to impaired fission or fusion has been shown to decrease mitochondrial translation, upregulating the UPR^mt^ and extending lifespan (Y. J. Liu et al., 2020).

Pharmacological agents can similarly extend lifespan through targeting activators and inhibitors of the UPR^mt^. Antimycin, an inhibitor of mitochondrial ETC complex III, extends lifespan (Dillin et al., 2002). Likewise, Metolazone, a blocker of Na + -Cl− cotransporters, including NKCC-1, also prolongs lifespan by activating the UPR^mt^. However how NKCC-1 activates the UPR^mt^ in *C. elegans* is still unclear (Ito et al., 2021). Another study has shown that NAD^+^ level decreases with age in *C. elegans*, and restoration of NAD^+^ levels with NAD + boosters increases *sir-2.1* (sirtuin homolog) activity, which in turn improves lifespan through activation of the UPR^mt^ (Mouchiroud et al., 2013). In addition, the mitochondrial chaperone prohibitin is an important part of the UPR^mt^ in longevity regulation (Gatsi et al., 2014). Interestingly, depletion of prohibitin, which induces UPR^mt^, shortens lifespan in wild type worms whereas this depletion extends lifespan in metabolically compromised worms (Artal-Sanz and Tavernarakis, 2009; Gatsi et al., 2014).

The lifespan extension afforded by the UPR^mt^ relies, at least in part, on cell non-autonomous interactions between different tissues. It has been shown that neuronal induction of UPR^mt^ by the accumulation of expanded-polyQ aggregates leads to UPR^mt^ induction in the intestine, and depends on the neuronal release of serotonin and long-range Wnt signaling pathway (Berendzen et al., 2016; Q.; Zhang et al., 2018). In addition to the nervous system, other tissues can also communicate their proteostasis status and induce the UPR^mt^ in distal tissues (Calculli et al., 2021). For instance, aggregation of the germ granule component PGL-1 triggers intracellular changes in the mitochondrial network of *C. elegans* germline cells. In turn, the germline releases long-range WNT ligands that induce an overactivation of the UPR^mt^ in somatic tissues, promoting somatic mitochondrial fragmentation and aggregation of proteins linked with age-related neurodegenerative diseases such amyotrophic lateral sclerosis and Huntington’s (Calculli et al., 2021).

Beyond the UPR^mt^, other factors could be involved in mitohormesis. The most prominent example is the contribution of ROS towards aging in respect to oxidative damage and regulatory roles. The Harman Free Radical Theory of Aging postulates that cellular aging is driven by the formation of mitochondrial ROS. These ROS induce a damage to distinct components of the cell, including DNA, the proteome, and the mitochondria itself. This has been challenged by studies in *C. elegans*, which have found that lifespan can be decoupled from oxidative damage or oxidant sensitivity. In fact, it has been proposed that low levels of ROS may be beneficial for longevity, where ROS could potentially act as signaling molecules that promote mitohormesis (Ristow and Schmeisser, 2014). For example, it has been reported that deletion of the mitochondrial superoxide dismutase *sod-2* increases ROS production, yet prolongs lifespan despite the increased levels of oxidative damaged proteins (Van Raamsdonk and Hekimi, 2009). Moreover, worms treated with low amounts of the oxidant reagent paraquat also live longer (Yang and Hekimi, 2010). One study showed that a mild reduction of respiration extends longevity through ROS mediated activation of the hypoxia-inducible factor HIF-1 (Lee et al., 2010). Glucose restriction also leads to the formation of ROS due to enhanced mitochondrial respiration, prolonging lifespan in *C. elegans* (Schulz et al., 2007). The knockdown of the hydroxylase *clk-1* was also found to enhance longevity despite elevated levels of ROS production (Lakowski and Hekimi, 1996; Lee et al., 2010) Together, these studies indicate that the role of ROS in mitohormesis and aging is less clear as previously thought, and further demonstrate possible roles of ROS as both signal molecules and cellular stress factors. Further studies are required to fully assess the contribution of ROS and oxidative damage towards organism health and longevity. Nevertheless, studies in *C. elegans* support that the UPR^mt^ can be a powerful promoter of longevity, and a promising therapeutic target for pharmacological intervention.

## 7 Conclusion

Ensuring proteome integrity requires tight regulation and crosstalk of distinct components of the proteostasis network from translation to degradation. However, with age, the burden of misfolded proteins exceeds the capacity of cells to maintain proper proteome integrity, leading to disruptions in cellular function. Cumulative evidence using *C. elegans* as a model organism has highlighted the important role of the proteostasis network in longevity regulation as well as the onset of age-related disease regulation. These studies in *C. elegans* have also provided invaluable information about the regulation of distinct proteostasis nodes at the organismal level, including their regulation by cell non-autonomous mechanisms that can be crucial to find novel therapeutic targets to delay age-related diseases in humans.
